# Integrated Information in the Spiking–Bursting Stochastic Model

**DOI:** 10.3390/e22121334

**Published:** 2020-11-24

**Authors:** Oleg Kanakov, Susanna Gordleeva, Alexey Zaikin

**Affiliations:** 1 Faculty of Radiophysics, Lobachevsky State University of Nizhny Novgorod, 603950 Nizhny Novgorod, Russia; okanakov@rf.unn.ru; 2 Institute of Biology and Biomedicine, Lobachevsky State University of Nizhny Novgorod, 603950 Nizhny Novgorod, Russia; gordleeva@neuro.nnov.ru; 3 Center for Technologies in Robotics and Mechatronics Components, Innopolis University, 420500 Innopolis, Russia; 4 Institute of Information Technology, Mathematics and Mechanics, Lobachevsky State University of Nizhny Novgorod, 603950 Nizhny Novgorod, Russia; 5 Institute for Women’s Health and Department of Mathematics, University College London, London WC1E 6BT, UK; 6 Centre for Analysis of Complex Systems, Sechenov University, 119991 Moscow, Russia

**Keywords:** integrated information, discrete-state stochastic model, computational biology, neuron-astrocyte networks

## Abstract

Integrated information has been recently suggested as a possible measure to identify a necessary condition for a system to display conscious features. Recently, we have shown that astrocytes contribute to the generation of integrated information through the complex behavior of neuron–astrocyte networks. Still, it remained unclear which underlying mechanisms governing the complex behavior of a neuron–astrocyte network are essential to generating positive integrated information. This study presents an analytic consideration of this question based on exact and asymptotic expressions for integrated information in terms of exactly known probability distributions for a reduced mathematical model (discrete-time, discrete-state stochastic model) reflecting the main features of the “spiking–bursting” dynamics of a neuron–astrocyte network. The analysis was performed in terms of the empirical “whole minus sum” version of integrated information in comparison to the “decoder based” version. The “whole minus sum” information may change sign, and an interpretation of this transition in terms of “net synergy” is available in the literature. This motivated our particular interest in the sign of the “whole minus sum” information in our analytical considerations. The behaviors of the “whole minus sum” and “decoder based” information measures are found to bear a lot of similarity—they have mutual asymptotic convergence as time-uncorrelated activity increases, and the sign transition of the “whole minus sum” information is associated with a rapid growth in the “decoder based” information. The study aims at creating a theoretical framework for using the spiking–bursting model as an analytically tractable reference point for applying integrated information concepts to systems exhibiting similar bursting behavior. The model can also be of interest as a new discrete-state test bench for different formulations of integrated information.

## 1. Introduction

Integrated information (II) [1,2,3,4] is a measure of internal information exchange in complex systems and it has recently attracted a lot of interest, because initially it was proposed to quantify consciousness [5]. Despite the fact that this initial aim is still a matter of research and debate [6,7,8,9], the II concept itself is by now a widely acknowledged tool in the field of complex dynamics analysis [10,11,12]. The general concept gave rise to specific “empirical” formalizations of II [13,14,15,16] aimed at computability from empirical probability distributions based on real data. For a systematic taxonomy of II measures, see [17], and a comparative study of empirical II measures in application to Gaussian autoregressive network models has been recently done in [18].

Our recent study [19] addressed the role of astrocytic regulation of neurotransmission [20,21,22] in generating positive II via small networks of brain cells—neurons and astrocytes. Empirical “whole minus sum” II, as defined in [13], was calculated in [19] from the time series produced by a biologically realistic model of neuro-astrocytic networks. A simplified, analytically tractable stochastic “spiking–bursting” model (in complement to the realistic one) was designed to describe a specific type of activity in neuro-astrocytic networks which manifests itself as a sequence of intermittent system-wide excitations of rapid pulse trains (“bursts”) on the background of random “spiking” activity in the network [23,24]. The spiking–bursting model is a discrete-time, discrete-state stochastic process which mimics the main features of this behavior. The model was successfully used in [19] to produce semi-analytical estimates of II in good agreement with direct computation of II from time series of the biologically realistic network model. We have suggested a possible explanation that a generation of positive II was the reason why mammal brain evolved to develop an astrocyte network to overlap with a network of neurons, but, still, it remained unclear what are the underlying mechanisms driving a complex neural behavior to generate positive II. In this paper we address this challenging question.

The present study aims at creating a theoretical formalism for using the spiking–bursting model of [19] as an analytically tractable reference point for applying integrated information concepts to systems exhibiting similar bursting behavior (in particular, to other neuron–astrocyte networks). The analytical treatment is based on exact and asymptotic expressions for integrated information in terms of exactly known probability distributions for the spiking–bursting model. The model is constructed as the simplest possible (although essentially non-Gaussian) to reflect the features of neuron–astrocyte network dynamics which lead to generating positive II. We also aim at extending the knowledge of comparative features of different empirical II measures, which are currently available mainly in application to Gaussian autoregressive models [17,18], by applying two such measures [13,16] to our discrete-state model.

In Section 2 and Section 3 we specify the definitions of the II measures used and the model. Specific properties of the model which lead to redundancy in its parameter set are addressed in Section 4. In Section 5 we provide an analytical treatment for the empirical “whole minus sum” [13] version of II in application to our model. This choice among other empirical II measures is inherited from the preceding study [19] and is in part due to its easy analytical tractability, and also due to its ability to change sign, which naturally identifies a transition point in the parameter space. This property may be considered a violation of the natural non-negativeness requirement for II [16]; on the other hand, the sign of the “whole minus sum” information has been given interpretation in terms of “net synergy” [25] as a degree of redundancy in the evolution of a system [18]. In this sense this transition may be viewed as a useful marker in its own right in the tool-set of measures for complex dynamics. This motivates our particular focus on identifying the sign transition of the “whole minus sum” information in the parameter space of the model. We also identify a scaling of II with a small parameter which determines time correlations in the bursting (astrocytic) subsystem.

In Section 6 we compare the outcome of the “whole minus sum” II measure [13] to that of the “decoder based” measure $\Phi^{*}$, which was specifically designed in [16] to satisfy the non-negativeness property. We compute $\Phi^{*}$ directly by definition from known probability distributions of the model. Despite their inherent difference consisting in changing or not changing sign, the two compared measures are shown to bear similarities in their dependence upon model parameters, including the same scaling with the time correlation parameter.

## 2. Definition of II Measures in Use

The empirical “whole minus sum” version of II is formulated according to [13] as follows. Consider a stationary stochastic process ξ(t) (binary vector process), whose instantaneous state is described by *N* binary digits (bits), each identified with a node of the network (neuron). The full set of *N* nodes (“system”) can be split into two non-overlapping non-empty subsets (“subsystems”) *A* and *B*; such a splitting is referred to as bipartition AB. Denote by x=ξ(t) and y=ξ(t+τ) two states of the process separated by a specified time interval τ≠0. The states of the subsystems are denoted as $x_{A}$, $x_{B}$, $y_{A}$, $y_{B}$.

Mutual information between *x* and *y* is defined as

(1) $$I_{xy}=H_{x}+H_{y}-H_{xy},$$

where

(2) $$H_{x}=-\sum_{x}p\left(x\right)\log_{2}p\left(x\right)$$

is entropy (base 2 logarithm gives result in bits); summation is hereinafter assumed to be taken over the whole range of the index variable (here *x*), $H_{y}=H_{x}$, due to assumed stationarity.

Next, a bipartition AB is considered, and “effective information” $\Phi_{\mathrm{eff}}$ as a function of the particular bipartition is defined as

(3) $$\Phi_{\mathrm{eff}}\left(AB\right)=I_{xy}-I_{x_{A},y_{A}}-I_{x_{B},y_{B}}.$$

Finally, “whole minus sum” II denoted as Φ is defined as effective information calculated for a specific bipartition $AB^{\mathrm{MIB}}$ (“minimum information bipartition”) which minimizes specifically normalized effective information:

(4a) $$\begin{array}{c} \Phi =\Phi_{\mathrm{eff}}\left(AB^{\mathrm{MIB}}\right), \end{array}$$

(4b) $$\begin{array}{c} AB^{\mathrm{MIB}}={\mathrm{argmin}}_{AB}\left[\frac{\Phi_{\mathrm{eff}}\left(AB\right)}{\min\{H\left(x_{A}\right),H\left(x_{B}\right)\}}\right]. \end{array}$$

Note that this definition prohibits positive II, whenever $\Phi_{\mathrm{eff}}$ turns out to be zero or negative for at least one bipartition AB.

We compare the result of the “whole minus sum” effective information (3) to the “decoder based” information measure $\Phi^{*}$, which is modified from its original formulation of [16] by setting the logarithms base to 2 for consistency:

(5a) $$\Phi^{*}\left(AB\right)=I_{xy}-I_{xy}^{*}\left(AB\right),$$

where

(5b) $$I_{xy}^{*}\left(AB\right)=\max_{\beta }\left[-\sum_{y}p\left(y\right)\log_{2}\sum_{x}p\left(x\right)q_{AB}{\left(y|x\right)}^{\beta }+\sum_{xy}p\left(xy\right)\log_{2}q_{AB}{\left(y|x\right)}^{\beta }\right],$$

(5c) $$q_{AB}\left(y|x\right)=p\left(y_{A}|x_{A}\right)p\left(y_{B}|x_{B}\right)=\frac{p\left(x_{A}y_{A}\right)p\left(x_{B}y_{B}\right)}{p\left(x_{A}\right)p\left(x_{B}\right)}.$$

## 3. Spiking–Bursting Stochastic Model

**Figure 1:**
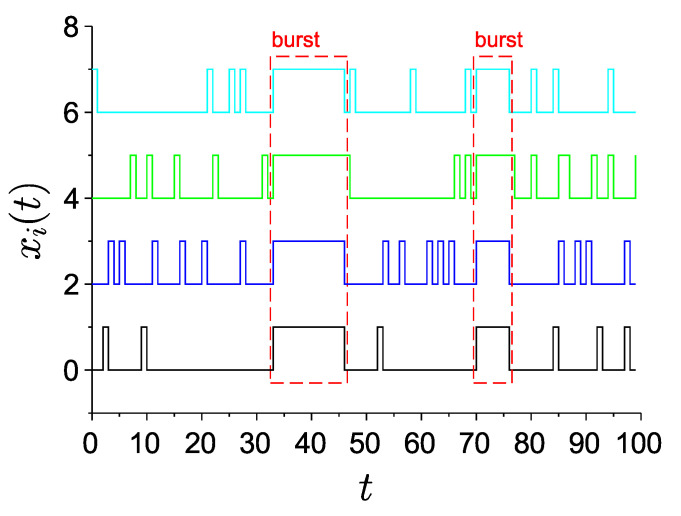
Typical time traces of the spiking–bursting model containing N=4 nodes (“neurons”) in discrete time. Plots for different neurons are shown with different constant shifts along the ordinate axis. Two bursts (marked) and background uncorrelated spiking dynamics are visible.

Physiologically, spikes are short (about 1 millisecond in duration) pulses of voltage (action potential) across the neuronal membrane. Bursts are rapid sequences of spikes. The main feature of the neuron–astrocyte network model in [19] is the presence of network-wide coordinated bursts, when all neurons are rapidly spiking in the same time window. Such bursts are coordinated by the astrocytic network and occur on the background of weakly correlated spiking activity of individual neurons. The spiking–bursting model was suggested in [19] as the simplest mathematical description of this behavior. In this model, time is discretized into small bins, and neurons are represented by binary digits taking on values 0 or 1, denoting the absence or the presence of at least one spike within the specific time bin. Respectively, a network-wide burst is represented by a time interval during which all neurons are locked at value 1 (which corresponds to a train of rapid spiking in the underlying biological system). The idea behind the model is illustrated by the graphical representation of its typical time evolution, as shown in Figure 1. The graphs of the model dynamics can be seen as envelopes of respective time recordings of membrane voltage in actual neurons: each short rectangular pulse of the model is assumed to correspond to at least one narrow spike of voltage, and a prolonged pulse (several discrete time bins in duration) represents a spike train (burst).

Mathematically, this “spiking–bursting” model is a stochastic model, which produces a binary vector valued, discrete-time stochastic process. In keeping with [19], the model is defined as a combination M={V,S} of a time-correlated dichotomous component *V* which turns on and off system-wide bursting (that mimics global bursting of a neuronal network, when each neuron produces a train of pulses at a high rate [19]), and a time-uncorrelated component *S* describing spontaneous (spiking) activity (corresponding to a background random activity in a neural network characterized by relatively sparse random appearance of neuronal pulses—spikes [19]) occurring in the absence of a burst. The model mimics the spiking–bursting type of activity which occurs in a neuro-astrocytic network, where the neural subsystem normally exhibits time-uncorrelated patterns of spiking activity, and all neurons are under the common influence of the astrocytic subsystem, which is modeled by the dichotomous component *V* and sporadically induces simultaneous bursting in all neurons. A similar network architecture with a “master node” spreading its influence on subordinated nodes was considered, for example, in [1] (Figure 4b therein).

The model is defined as follows. At each instance of (discrete) time the state of the dichotomous component can be either “bursting” with probability $p_{b}$, or “spontaneous” (or “spiking”) with probability $p_{s}=1-p_{b}$. While in the bursting mode, the instantaneous state of the resulting process x=ξ(t) is given by all ones: x=11..1 (further abbreviated as x=1). In cases of spiking, the state *x* is a (time-uncorrelated) random variate, which is described by a discrete probability distribution $s_{x}$ (where an occurrence of “1” in any bit is referred to as a “spike”), so that the resulting one-time state probabilities read

(6a) $$\begin{array}{cc} p(x\neq 1) & =p_{s}s_{x}, \end{array}$$

(6b) $$\begin{array}{cc} p(x=1) & =p_{1},\;p_{1}=p_{s}s_{1}+p_{b}, \end{array}$$

where $s_{1}$ is the probability of spontaneous occurrence of x=1 (hereafter referred to as a system-wide simultaneous spike) in the absence of a burst. (In a real network, “simultaneous” implies occurring within the same time discretization bin [19]).

To describe two-time joint probabilities for x=ξ(t) and y=ξ(t+τ), consider a joint state xy which is a concatenation of bits in *x* and *y*. The spontaneous activity is assumed to be uncorrelated in time, which leads to the factorization

(7) $$s_{xy}=s_{x}s_{y}.$$

The time correlations of the dichotomous component are described by a 2×2 matrix

(8) $$p_{q\in \{s,b\},r\in \{s,b\}}=\left(\begin{array}{cc} p_{ss} & p_{sb}\\ p_{bs} & p_{bb} \end{array}\right)$$

whose components are joint probabilities to observe the respective spiking (index “*s*”) and/or bursting (index “*b*”) states in *x* and *y*. (In a neural network these correlations are conditioned by burst duration [19]; e.g., if this (in general, random) duration mostly exceeds τ, then the correlation is positive.) The probabilities obey $p_{sb}=p_{bs}$ (due to stationarity), $p_{b}=p_{bb}+p_{sb}$, $p_{s}=p_{ss}+p_{sb}$, thereby allowing one to express all one- and two-time probabilities describing the dichotomous component in terms of two independent quantities, which for example, can be a pair $\left\{p_{s},p_{ss}\right\}$; then

(9a) $$\begin{array}{cc} p_{sb} & =p_{bs}=p_{s}-p_{ss}, \end{array}$$

(9b) $$\begin{array}{cc} p_{bb} & =1-(p_{ss}+2p_{sb}), \end{array}$$

or $\left\{p_{b},\rho \right\}$ as in [19], where ρ is the Pearson correlation coefficient defined by

(10) $$p_{sb}=p_{s}p_{b}\left(1-\rho \right).$$

In Section 4 we justify the use of another effective parameter ϵ (13) instead of ρ to determine time correlations in the dichotomous component.

The two-time joint probabilities for the resulting process are then expressed as

(11a) $$\begin{array}{c} \begin{array}{cc} p(x\neq 1,y\neq 1) & =p_{ss}s_{x}s_{y},\\ p(x\neq 1,y=1) & =\pi s_{x},\;p\left(x=1,y\neq 1\right)=\pi s_{y},\\ p(x=1,y=1) & =p_{11}, \end{array} \end{array}$$

(11b) $$\begin{array}{c} \pi =p_{ss}s_{1}+p_{sb},\;p_{11}=p_{ss}s_{1}^{2}+2p_{sb}s_{1}+p_{bb}. \end{array}$$

Note that the above notation can be applied to any subsystem instead of the whole system (with the same dichotomous component, as it is system-wide anyway).

The mentioned probabilities can be interpreted in terms of the underlying biological system as follows (see details in [19]): $p_{b}$ is the probability of observing the astrocytic subsystem in the excited (high calcium concentration) state, which induces global bursting activity in all neurons, within a specific time discretization bin; $p_{bb}$ is the probability of observing the mentioned state in two time bins separated by the time lag τ, and ρ is the respective time-delayed Pearson correlation coefficient of the astrocytic activity; $s_{x}$ is the probability of observing a specific spatial pattern of spiking *x* within one time bin in spontaneous neuronal activity (in the absence of an astrocyte-induced burst), and in particular $s_{1}$ is the probability that all neurons fire a spike within one time bin in spontaneous activity. In this sense $s_{1}$ measures the overall strength of spontaneous activity of the neuronal subsystem. When spiking activity is independent across neurons, the set of parameters $\left\{s_{1},p_{b},\rho \right\}$ fully determines the “whole minus sum” II in the spiking–bursting model. In [19] these parameters were fitted to match (in the least-squares sense) the two-time probability distribution (11) to the respective “empirical” (numerically obtained) probabilities for the biologically realistic model of the neuron–astrocyte network. This fitting produced the dependence of the spiking–bursting parameters $\left\{s_{1},p_{b},\rho \right\}$ upon the biological parameters; see Figure 7 in [19].

## 4. Model Parameter Scaling

The spiking–bursting stochastic model, as described in Section 3, is redundant in the following sense. In terms of the model definition, there are two distinct states of the model which equally lead to observing the same one-time state of the resultant process with 1s in all bits: firstly—a burst, and secondly—a system-wide simultaneous spike in the absence of a burst, which are indistinguishable by one-time observations. Two-time observations reveal a difference between system-wide spikes on one hand and bursts on the other, because the latter are assumed to be correlated in time, unlike the former. That said, the “labeling” of bursts versus system-wide spikes exists in the model (by the state of the dichotomous component), but not in the realizations. Proceeding from the realizations, it must be possible to relabel a certain fraction of system-wide spikes into bursts (more precisely, into a time-uncorrelated portion thereof). Such relabeling would change both components of the model {V,S} (dichotomous and spiking processes), in particular, diluting the time correlations of bursts, without changing the actual realizations of the resultant process. This implies the existence of a transformation of model parameters which keeps realizations (i.e., the stochastic process as such) invariant. The derivation of this transformation is presented in Appendix A and leads to the following scaling.

(12a) $$\begin{array}{cc} s_{x\neq 1} & =\alpha s_{x\neq 1}^{\prime }, \end{array}$$

(12b) $$\begin{array}{cc} 1-s_{1} & =\alpha (1-s_{1}^{\prime }), \end{array}$$

(12c) $$\begin{array}{cc} p_{s^{\prime }} & =\alpha p_{s}, \end{array}$$

(12d) $$\begin{array}{cc} p_{s^{\prime }s^{\prime }} & =\alpha^{2}p_{ss}, \end{array}$$

where α is a positive scaling parameter, and all other probabilities are updated according to Equation (9).

The mentioned invariance in particular implies that any characteristic of the process must be invariant to the scaling (12a–d). This suggests a natural choice of a scaling-invariant effective parameter ϵ defined by

(13) $$p_{ss}=p_{s}^{2}\left(1+\epsilon \right)$$

to determine time correlations in the dichotomous component. In conjunction with a second independent parameter of the dichotomous process, for which a straightforward choice is $p_{s}$, and with full one-time probability table for spontaneous activity $s_{x}$, these constitute a natural full set of model parameters $\left\{s_{x},p_{s},\epsilon \right\}$.

The two-time probability table (8) can be expressed in terms of $p_{s}$ and ϵ by substituting Equation (13) into Equation (9):

(14) $$p_{q\in \{s,b\},r\in \{s,b\}}=\left(\begin{array}{cc} p_{s}^{2}+\epsilon p_{s}^{2} & p_{s}p_{b}-\epsilon p_{s}^{2}\\ p_{s}p_{b}-\epsilon p_{s}^{2} & p_{b}^{2}+\epsilon p_{s}^{2} \end{array}\right).$$

The requirement of non-negativeness of probabilities imposes simultaneous constraints

(15a) $$\begin{array}{c} \epsilon \geq -1 \end{array}$$

and

(15b) $$\begin{array}{c} p_{s}\leq p_{s\max}=\left\{\begin{array}{cc} \frac{1}{1+\epsilon }\left(1-\sqrt{\left|\epsilon \right|}\right) & \mathrm{if}\;-1\leq \epsilon <0,\\ \frac{1}{1+\epsilon } & \mathrm{if}\;\epsilon \geq 0, \end{array}\right. \end{array}$$

or equivalently,

(16) $$-\epsilon_{\max}^{2}\leq \epsilon \leq \epsilon_{\max}=\frac{p_{b}}{p_{s}}.$$

Comparing the off-diagonal term $p_{sb}$ in (14) to the definition of the Pearson’s correlation coefficient ρ in (10), we get

(17) $$\epsilon =\rho \;\frac{p_{b}}{p_{s}}=\rho \;\epsilon_{\max};$$

thus, the sign of ϵ has the same meaning as that of ρ. Hereinafter we limit ourselves to non-negative correlations ϵ≥0.

## 5. Analysis of the Empirical “Whole Minus Sum” Measure for the Spiking–Bursting Process

In this Section we analyze the behavior of the “whole minus sum” empirical II [13] defined by Equations (3) and (4) for the spiking–bursting model in dependence of the model parameters, particularly focusing on its transition from negative to positive values.

### 5.1. Expressing the “Whole Minus Sum” Information

Mutual information $I_{xy}$ for two time instances *x* and *y* of the spiking–bursting process is expressed by inserting all one- and two-time probabilities of the process according to (6), (11) into the definition (1), (2). The full derivation is given in Appendix B and leads to an expression which was used in [19]

(18) $$I_{xy}=2\left(1-s_{1}\right)\left\{p_{s}\right\}+2\left\{p_{1}\right\}-{\left(1-s_{1}\right)}^{2}\left\{p_{ss}\right\}-2\left(1-s_{1}\right)\left\{\pi \right\}-\left\{p_{11}\right\},$$

where we denote for compactness

(19) $$\left\{q\right\}=-q\log_{2}q.$$

We exclude from further consideration the following degenerate cases which automatically give $I_{xy}=0$ by definition (1):

(20) $$s_{1}=1,\;\mathrm{or}\;p_{s}=0,\;\mathrm{or}\;p_{s}=1,\;\mathrm{or}\;\rho =\epsilon =0,$$

where the former two correspond to a deterministic “always 1” state for which all entropies in (1) are zero, and the latter two produce no predictability, which implies $H_{xy}=H_{x}+H_{y}$.

The particular case $s_{1}=0$ in (18) reduces to

(21a) $$I_{xy}{|}_{s_{1}=0}=2\left(\left\{p_{s}\right\}+\left\{p_{b}\right\}\right)-\left(\left\{p_{ss}\right\}+2\left\{p_{sb}\right\}+\left\{p_{bb}\right\}\right),$$

which coincides with mutual information for the dichotomous component taken alone and can be seen as a function of just two independent parameters of the dichotomous component, for which we chose $p_{s}$ and ϵ as suggested in Section 4. Using the expressions for the two-time probabilities (14), we rewrite (21a) in the form

(21b) $$\begin{array}{cc} I_{xy}{|}_{s_{1}=0} & =2\left(\left\{p_{s}\right\}+\left\{p_{b}\right\}\right)-\left(\left\{p_{s}^{2}+\epsilon p_{s}^{2}\right\}+2\left\{p_{s}p_{b}-\epsilon p_{s}^{2}\right\}+\left\{p_{b}^{2}+\epsilon p_{s}^{2}\right\}\right),\;\mathrm{where}\;p_{b}=1-p_{s},\\ \; & =I_{0}\left(p_{s},\epsilon \right). \end{array}$$

**Figure 2:**
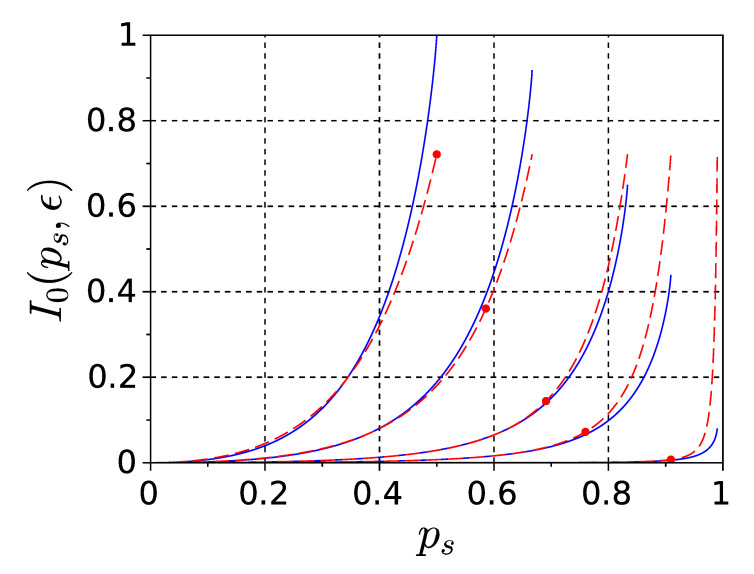
Blue solid lines—plots of $I_{0}\left(p_{s},\epsilon \right)$ versus $p_{s}$ varied from 0 to $p_{s\max}$ as per (15), at ϵ=0.01, 0.1, 0.2, 0.5, 1 (from right to left). Function $I_{0}\left(p_{s},\epsilon \right)$ is a universal single function of two arguments, which is explicitly expressed in elementary functions in (21b), and allows one to express mutual information (18) and effective information (3) in terms of the model parameters. Red dashed lines—approximation (35). Red dots—upper bounds of approximation applicability range (36c).

Expression (21b) explicitly defines a function $I_{0}\left(p_{s},\epsilon \right)$, which turns out to be a universal function allowing one to express mutual information (18) and effective information (3) in terms of the model parameters, as we show below. Typical plots of $I_{0}\left(p_{s},\epsilon \right)$ versus $p_{s}$ at several fixed values of ϵ are shown with blue solid lines in Figure 2.

The formula (18) can be recovered back from (21a,b) by virtue of the scaling (12a–d), by assuming $s_{1}^{\prime }=0$ in (21b) and substituting the corresponding scaled value $p_{s^{\prime }}=\left(1-s_{1}\right)p_{s}$ as per (12c) in place of the first argument of function $I_{0}\left(p_{s^{\prime }},\epsilon \right)$ defined in (21b), while parameter ϵ remains invariant to the scaling. This produces a simplified expression

(22) $$I_{xy}=I_{0}\left(\left(1-s_{1}\right)p_{s},\epsilon \right),$$

which is exactly equivalent to (18) for any $s_{1}$. We emphasize that hereinafter expressions containing $I_{0}\left(\cdot ,\cdot \right)$—(22), (23), (30b), etc.—imply that $p_{s}$ in (21b) must be substituted with the actual first argument of $I_{0}\left(\cdot ,\cdot \right)$, e.g., by $\left(1-s_{1}\right)p_{s}$ in (22). The same applies when the approximate expression for $I_{0}\left(\cdot \right)$ (35) is used.

Given a bipartition AB (see Section 2), this result is applicable as well to any subsystem *A* (*B*), with $s_{1}$ replaced by $s_{A}$ ($s_{B}$) which denote the probability of a subsystem-wide simultaneous spike $x_{A}=1$ ($x_{B}=1$) in the absence of a burst, and with same parameters of the dichotomous component (here $p_{s}$, ϵ). Then effective information (3) is expressed as

(23) $$\Phi_{\mathrm{eff}}=I_{0}\left(\left(1-s_{1}\right)p_{s},\epsilon \right)-I_{0}\left(\left(1-s_{A}\right)p_{s},\epsilon \right)-I_{0}\left(\left(1-s_{B}\right)p_{s},\epsilon \right).$$

Hereafter in this section we assume the independence of spontaneous activity across the network nodes (neurons), which implies

(24) $$s_{A}s_{B}=s_{1},$$

then (23) turns into

(25a) $$\Phi_{\mathrm{eff}}=f\left(s_{A}\right),$$

where

(25b) $$f\left(s\right)=I_{0}\left(\left(1-s_{1}\right)p_{s},\epsilon \right)-I_{0}\left(\left(1-s\right)p_{s},\epsilon \right)-I_{0}\left(\left(1-s_{1}/s\right)p_{s},\epsilon \right).$$

Essentially, according to (25a,b), the function f(s) shows the dependence of effective information $\Phi_{\mathrm{eff}}$ upon the choice of the bipartition, which is characterized by the value of $s_{A}=s$ (if *A* is any non-empty subsystem, then $s_{A}$ is defined as the probability of spontaneous occurrence of 1s in all bits in *A* in the same instance of the discrete time), while the function parameter $s_{1}$ determines the intensity of spontaneous spiking activity. Note that the function $I_{0}\left(\cdot ,\cdot \right)$ in (21b) is defined only when the first argument is in the range (0,1); thus, the definition domain of f(s) in (25b) is

(26) $$s_{1}<s<1.$$

### 5.2. Determining the Sign of the “Whole Minus Sum” Information

According to (4), the necessary and sufficient condition for the “whole minus sum” empirical II to be positive is the requirement that $\Phi_{\mathrm{eff}}$ be positive for any bipartition AB. Due to (25a,b), this requirement can be written in the form

(27) $$\min_{s\in \{s_{A}\}}f\left(s\right)>0,$$

where $\left\{s_{A}\right\}$ is the set of $s_{A}$ values for all possible bipartitions AB.

Expanding the set of *s* in (27) to the whole definition domain of f(s) (26) leads to a sufficient (generally, stronger) condition for positive II

(28) $$f\left(s\right)>0\;\mathrm{for}\;\mathrm{all}\;s\in \left(s_{1},1\right).$$

**Figure 3:**
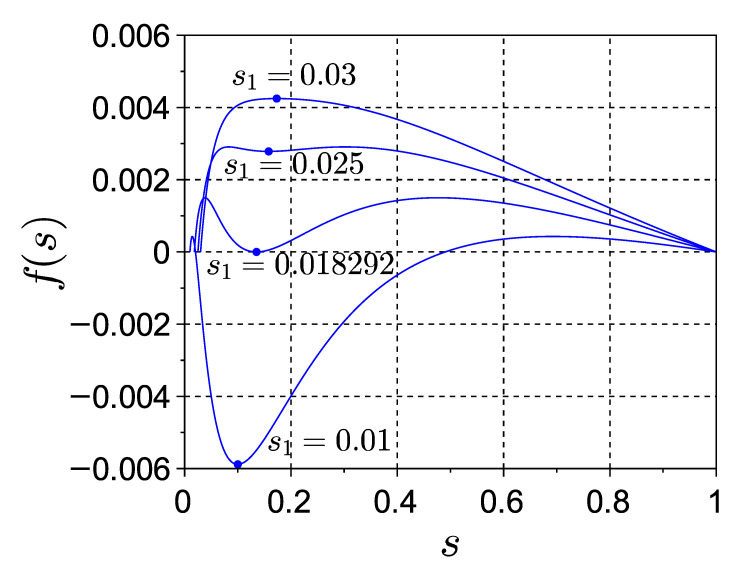
Plots of f(s) on $s_{1}<s<1$ for several values of $s_{1}$ (as indicated) at $p_{s}=0.7$, ϵ=0.1. According to (25a,b), f(s) shows the dependence of effective information $\Phi_{\mathrm{eff}}$ upon the choice of the bipartition AB, which is characterized by the value of $s_{A}=s$, while the function parameter $s_{1}$ determines the intensity of spontaneous spiking activity. For each value of $s_{1}$, the extremum $\left(\sqrt{s_{1}},f\left(\sqrt{s_{1}}\right)\right)$ is indicated with a dot.

Note that f(s) by definition (25b) satisfies $f\left(s=s_{1}\right)=f\left(s=1\right)=0$, $f^{\prime }\left(s=s_{1}\right)>0$ and (due to the invariance to mutual renaming of subsystems *A* and *B*) $f\left(s_{1}/s\right)=f\left(s\right)$. (All mentioned properties and subsequent reasoning can be observed in Figure 3, which shows a few sample plots of f(s)). The latter symmetry implies that the quantity of extrema of f(s) on $s\in (s_{1},1)$ must be odd, one of them always being at $s=\sqrt{s_{1}}$. If the latter is the only extremum, then it is a positive maximum, and (28) is thus fulfilled automatically. In case of three extrema, $f(\sqrt{s_{1}})$ is a minimum, which can change sign. In both these cases the condition (28) is equivalent to the requirement

(29) $$f(\sqrt{s_{1}})>0,$$

which can be rewritten as

(30a) $$g(s_{1})>0,$$

where

(30b) $$g\left(s_{1}\right)=f\left(\sqrt{s_{1}}\right)=I_{0}\left(\left(1-s_{1}\right)p_{s},\epsilon \right)-2I_{0}\left(\left(1-\sqrt{s_{1}}\right)p_{s},\epsilon \right).$$

The reasoning above essentially reduces the problem of determining the sign of II to determining the sign of the extremum $f(\sqrt{s_{1}})$.

The equivalence of (29) to (28) could be broken if f(s) had five or more extrema. As suggested by the numerical calculation on a grid of $p_{s}\in \left[0.01,0.99\right]$ and ρ∈[0.01,1], both with step 0.01, this exception never holds, although we did not prove this rigorously. Based on the reasoning above, in the following we assume the equivalence of (29) (and (30)) to (28).

A typical scenario of transformations of f(s) with the change of $s_{1}$ is shown in Figure 3. Here the extremum $f(\sqrt{s_{1}})$ (shown with a dot) transforms with the decrease of $s_{1}$ from a positive maximum into a minimum, which in turn decreases from positive through zero into negative values.

Note that by construction, the function $g(s_{1})$ defined in (30b) expresses effective information $\Phi_{\mathrm{eff}}$ from (3) for the hypothetic bipartition characterized by $s_{A}=s_{B}=\sqrt{s_{1}}$, which may or may not exist in the actual particular system. If such “symmetric” bipartition exists, then the value $s_{A}=\sqrt{s_{1}}$ belongs to the set $\left\{s_{A}\right\}$ in (27), which implies that (29) (same as (30)) is equivalent not only to (28), but also to the necessary and sufficient condition (27). Otherwise, (28) (equivalently, (29) or (30)), formally being only sufficient, still may produce a good estimate of the necessary and sufficient condition in cases when $\left\{s_{A}\right\}$ contains values which are close to $\sqrt{s_{1}}$ (corresponding to nearly symmetric partitions, if such exist).

**Figure 4:**
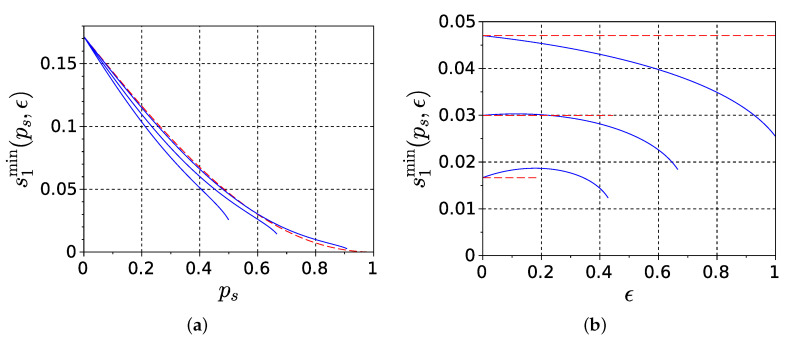
Graphs of threshold $s_{1}^{\min}$ determining the minimal sufficient intensity of spontaneous neuronal spiking activity for positive II. (**a**) Blue solid lines—plots of $s_{1}^{\min}\left(p_{s},\epsilon \right)$ versus $p_{s}$ varied from 0 to $p_{s\max}$ as per (15), at ϵ=0.1, 0.5, 1 (from right to left). Red dashed line—plot of the asymptotic formula (38). (**b**) Blue solid lines—plots of $s_{1}^{\min}\left(p_{s},\epsilon \right)$ versus ϵ varied from 0 to $\epsilon_{\max}$ as per (16), at $p_{s}=0.5$, 0.6, 0.7 (from top to bottom). Vertical position of red dashed lines is the result of (38), horizontal span denotes the estimated applicability range (36b).

Except for the degenerate cases (20), $g(s_{1})$ is negative at $s_{1}=0$

(31) $$g\left(s_{1}=0\right)=-I_{0}\left(p_{s},\epsilon \right)<0$$

and has a limit $g(s_{1}\to 1-0)\to +0$ ( −0 and +0 denote the left and right one-sided limits), because

(32) $$\lim_{s_{1}\to 1-0}\;\frac{I_{0}\left(\left(1-s_{1}\right)p_{s},\epsilon \right)}{2I_{0}\left(\left(1-\sqrt{s_{1}}\right)p_{s},\epsilon \right)}=2;$$

hence, $g(s_{1})$ changes sign at least once on $s_{1}\in \left(0,1\right)$. According to numerical evidence, we assume that $g(s_{1})$ changes sign exactly once on (0,1) without providing a rigorous proof for the latter statement (it was confirmed up to machine precision for each combination of $p_{s}\in \left[0.01,0.99\right]$ and ρ∈[0.01,1], both with step 0.01; also note that for the asymptotic case (38) this statement is rigorous). In line with the above, the solution to (30a) has the form

(33) $$s_{1}^{\min}\left(p_{s},\epsilon \right)<s_{1}<1,$$

where $s_{1}^{\min}\left(p_{s},\epsilon \right)$ is the unique root of $g(s_{1})$ on (0,1). Several plots of $s_{1}^{\min}\left(p_{s},\epsilon \right)$ versus $p_{s}$ at ϵ fixed and versus ϵ at $p_{s}$ fixed, which are obtained by numerically solving for the zero of $g(s_{1})$, are shown in Figure 4 with blue solid lines.

This result identifies a region in the parameter space of the model, where the “whole minus sum” information is positive. From the viewpoint of the underlying biological system, the quantity $s_{1}^{\min}$ determines the minimal sufficient intensity of spontaneous neuronal spiking activity for positive II. According to the result in Figure 4, within the assumption of independent spiking across the network (24), values $s_{1}≳0.17$ lead to positive II regardless of other parameter values, and this threshold decreases further when $p_{s}$ is increased, which implies decreasing the frequency of occurrence of astrocyte-activated global bursting $p_{b}=1-p_{s}$.

### 5.3. Asymptotics for Weak Correlations in Time

Further insight into the dependence of mutual information $I_{xy}$ (and, consequently, of $\Phi_{\mathrm{eff}}$ and II) upon parameters can be obtained by expanding the definition of $I_{0}\left(p_{s},\epsilon \right)$ in (21b) in powers of ϵ (limit of weak correlations in time), which yields

(34) $$I_{0}\left(p_{s},\epsilon \right)=\frac{1}{2\log2}{\left(\frac{p_{s}}{1-p_{s}}\right)}^{2}\cdot \epsilon^{2}+O\left(\epsilon^{3}\right).$$

Estimating the residual term (see details in Appendix C) indicates that the approximation by the leading term

(35) $$I_{0}\left(p_{s},\epsilon \right)\approx \frac{\epsilon^{2}}{2\log2}{\left(\frac{p_{s}}{1-p_{s}}\right)}^{2}$$

is valid when

(36a) $$\begin{array}{cc} \left|\epsilon \right| & \ll 1, \end{array}$$

(36b) $$\begin{array}{cc} \left|\epsilon \right| & \ll {\left(\frac{p_{b}}{p_{s}}\right)}^{2}\;=\epsilon_{\max}^{2}\;. \end{array}$$

Solving (36b) for $p_{s}$ rewrites it in the form of an upper bound for $p_{s}$

(36c) $$p_{s}<\frac{1}{1+\sqrt{\left|\epsilon \right|}}$$

(the use of “≪” sign is not appropriate in (36c), because this inequality does not imply a small ratio between its left-hand and right-hand parts). Note that the inequalities (36b), (36c) are not weaker than the formal upper bounds $\epsilon_{\max}$ in (16) and $p_{s\max}$ in (15) which arise from the definition of ϵ (13) due to the requirement of positive probabilities.

Approximation (35) is plotted in Figure 2 with red dashed lines along with corresponding upper bounds of approximation applicability range (36c) denoted by red dots (note that large ϵ violates (36a) anyway, thus in this case (36c) has no effect). Mutual information (35) scales with ϵ within range (36) as $\epsilon^{2}$ and vanishes with ϵ→0. The same holds for effective information (23). Since the normalizing denominator in (4b) contains one-time entropies which do not depend on ϵ at all, this scaling of $\Phi_{\mathrm{eff}}$ does not change the minimum information bipartition, finally implying that II also scales as $\epsilon^{2}$. That said, as factor $\epsilon^{2}$ does not affect the sign of $\Phi_{\mathrm{eff}}$, the lower bound $s_{1}^{\min}$ in (33) exists and is determined only by $p_{s}$ in this limit.

Substituting the approximation (35) for $I_{0}\left(\cdot ,\cdot \right)$ into the definition of $g(s_{1})$ in (30b) after simplifications reduces the equation $g(s_{1})=0$ to the following (see the comment below Equation (22)):

(37) $$p_{s}\left(\sqrt{2}-1\right)\;s_{1}-\sqrt{s_{1}}+\left(1-p_{s}\right)\left(\sqrt{2}-1\right)=0\;,$$

whose solution in terms of $s_{1}$ on $0<s_{1}<1$ equals $s_{1}^{\min}$, according to the reasoning behind Equation (33). Solving (37) as a quadratic equation in terms of $\sqrt{s_{1}}$ produces a unique root on (0,1), which yields

(38) $$s_{1}^{\min}\left(p_{s}\right){|}_{\epsilon \to 0}={\left(\frac{1-\sqrt{1-4p_{s}\left(1-p_{s}\right){\left(\sqrt{2}-1\right)}^{2}}}{2p_{s}\left(\sqrt{2}-1\right)}\right)}^{2}.$$

Result of (38) is plotted in Figure 4 with red dashed lines: in panel (a) as a function of $p_{s}$, and in panel (b) as horizontal lines whose vertical position is the result of (38), and horizontal span denotes the estimated applicability range (36b) (note that condition (36a) also applies, and becomes stronger than (36b) when $p_{s}<1/2$).

## 6. Comparison of Integrated Information Measures

In this Section we compare the outcome of two versions of empirical integrated information measures available in the literature, one being the “all-minus-sum” effective information $\Phi_{\mathrm{eff}}$ (3) from [13] which is used elsewhere in this study, and the other “decoder based” information $\Phi^{*}$ as introduced in [16] and expressed by Equations (5a–c). We calculate both measures by their respective definitions using the one- and two-time probabilities from Equations (6a,b) and (11a–d) for the spiking–bursting model with N=6 bits, assuming no spatial correlations among bits in spiking activity, with same spike probability *P* in each bit. In this case

(39) $$s_{x}=P^{m(x)}{\left(1-P\right)}^{N-m(x)},\;P=s_{1}^{\frac{1}{N}},$$

where m(x) is the number of ones in the binary word *x*.

We consider only a symmetric bipartition with subsystems *A* and *B* consisting of N/2=3 bits each. Due to the assumed equal spike probabilities in all bits and in the absence of spatial correlations of spiking, this implies complete equivalence between the subsystems. In particular, in the notations of Section 5 we get

(40) $$s_{1}=s_{A}s_{B},\;s_{A}=s_{B}=\sqrt{s_{1}}.$$

This choice of the bipartition is firstly due to the fact that the sign of effective information for this bipartition determines the sign of the resultant “whole minus sum” II (although the actual value of II is determined by the minimal information bipartition, which may be different). This has been established in Section 5 (see reasoning behind Equations (27)–(30) and further on); moreover, the function $g(s_{1})$ introduced in Equation (30b) expresses effective information for this particular bipartition

(41) $$\Phi_{\mathrm{eff}}\left(AB\right)=g\left(s_{1}\right),$$

thus the analysis of effective information sign in Section 5 applies to this symmetric bipartition.

Moreover, the choice of the symmetric bipartition is consistent with available comparative studies of II measures [18], where it was substantiated by the conceptual requirement that highly asymmetric partitions should be excluded [2], and by the lack of a generally accepted specification of minimum information bipartition; for further discussion, see [18].

**Figure 5:**
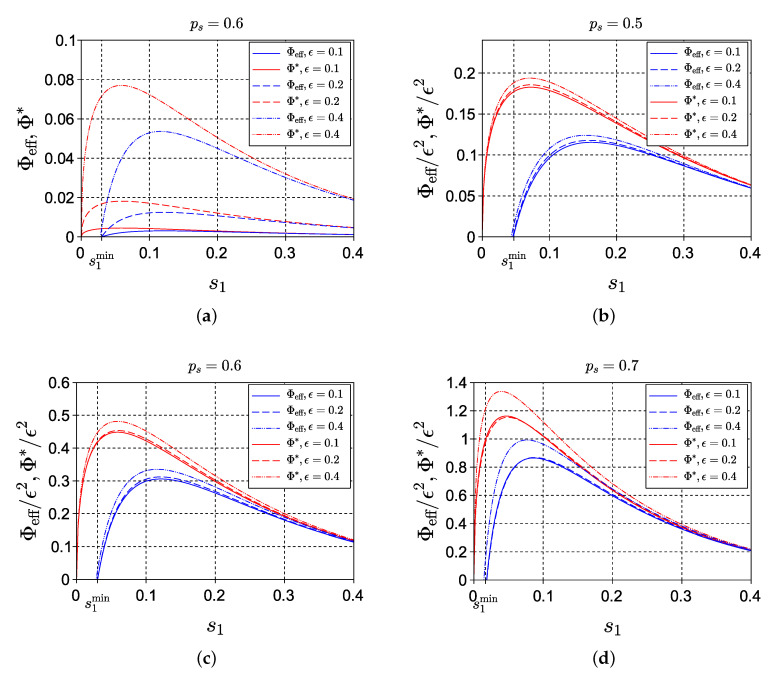
Comparison of two versions of empirical effective information for the symmetric bipartition—“whole-minus-sum” measure $\Phi_{\mathrm{eff}}$ (3) from [13] (blue lines) and “decoder based” information $\Phi^{*}$ (5) from [16] (red lines) versus spiking activity parameter $s_{1}$ at various fixed values of the bursting component parameters $p_{s}$ (indicated on top of the panels) and ϵ (indicated in the legends). Panel (**a**)—unnormalized values, panels (**b**–**d**)—normalized by $\epsilon^{2}$. Threshold $s_{1}^{\min}$ calculated according to (38) is shown in each panel with an additional vertical grid line.

We have studied the dependence of the mentioned effective information measures $\Phi_{\mathrm{eff}}$ and $\Phi^{*}$ upon spiking activity, which is controlled by $s_{1}$, at different fixed values of the parameters $p_{s}$ and ϵ characterizing the bursting component. Typical dependence of $\Phi_{\mathrm{eff}}$ and $\Phi^{*}$ upon $s_{1}$, taken at $p_{s}=0.6$ with several values of ϵ, is shown in Figure 5, panel (a).

The behavior of the “whole minus sum” effective information $\Phi_{\mathrm{eff}}$ (41) (blue lines in Figure 5) is found to agree with the analytical findings of Section 5:

- $\Phi_{\mathrm{eff}}$ transitions from negative to positive values at a certain threshold value of $s_{1}=s_{1}^{\min}$, which is well approximated by the formula (38) when ϵ is small, as required by (36a,b); the result of Equation (38) is indicated in each panel of Figure 5 by an additional vertical grid line labeled $s_{1}^{\min}$ on the abscissae axis—cf. Figure 4;
- $\Phi_{\mathrm{eff}}$ reaches a maximum on the interval $s_{1}^{\min}<s_{1}<1$ and tends to zero (from above) at $s_{1}\to 1$;
- $\Phi_{\mathrm{eff}}$ scales with ϵ as $\epsilon^{2}$, when (36a,b) hold.

To verify the scaling observation, we plot the scaled values of both information measures $\Phi_{\mathrm{eff}}/\epsilon^{2}$, $\Phi^{*}/\epsilon^{2}$ in the panels (b)–(d) of Figure 5 for several fixed values of $p_{s}$ and ϵ. Expectedly, the scaling fails at $p_{s}=0.7$, ϵ=0.4 in panel (d), as (36b) is not fulfilled in this case.

Furthermore, the “decoder based” information $\Phi^{*}$ (plotted with red lines in Figure 5) behaves mostly the same way, apart from being always non-negative (which was one of key motivations for introducing this measure in [16]). At the same time, the sign transition point $s_{1}^{\min}$ of the “whole minus sum” information associates with a rapid growth of the “decoder based” information. When $s_{1}$ is increased towards 1, the two measures converge. Remarkably, the scaling as $\epsilon^{2}$ is found to be shared by both effective information measures.

## 7. Discussion

In general, the spiking–bursting model is completely specified by the combination of a full single-time probability table $s_{x}$ (consisting of $2^{N}$ probabilities of all possible outcomes, where *N* is the number of bits) for the time-uncorrelated spontaneous activity, along with two independent parameters (e.g., $p_{s}$ and ϵ) for the dichotomous component. This combination is, however, redundant in that it admits a one-parameter scaling (12) which leaves the resultant stochastic process invariant.

Condition (30) was derived assuming that spiking activity in individual bits (i.e., nodes, or neurons) constituting the system is independent among the bits, which implies that the probability table $s_{x}$ is fully determined by *N* spike probabilities for individual nodes. The condition is formulated in terms of $p_{s}$, ϵ and a single parameter $s_{1}$ (system-wide spike probability) for the spontaneous activity, agnostic of the “internal structure” of the system, i.e., the spike probabilities for individual nodes. This condition provides that the “whole minus sum” effective information is positive for any bipartition, regardless of the mentioned internal structure. Moreover, in the limit (36) of weak correlations in time, the inequality (30a) can be explicitly solved in terms of $s_{1}$, producing the solution (33), (38).

In this way, the inequality (33) together with the asymptotic estimate (38) supplemented by its applicability range (36) specifies the region in the parameter space of the system, where the “whole minus sum” II is positive regardless of the internal system structure (sufficient condition). The internal structure (though still without spike correlations across the system) is taken into account by the necessary and sufficient condition (27) for positive II.

The mentioned conditions were derived under the assumption of absent correlation between spontaneous activity in individual bits (24). If correlation exists and is positive, then $s_{1}>s_{A}s_{B}$, or $s_{B}<s_{1}/s_{A}$. Then comparing the expressions for $\Phi_{\mathrm{eff}}$ (23) (general case) to (25) (space-uncorrelated case), and taking into account that $I_{0}\left(p_{s}\right)$ is an increasing function, we find $\Phi_{\mathrm{eff}}<f\left(s_{A}\right)$, cf. (25a). This implies that any necessary condition for positive II remains as such. Likewise, in the case of negative correlations we get $\Phi_{\mathrm{eff}}>f\left(s_{A}\right)$, implying that a sufficient condition remains as such.

## 8. Conclusions

The present study substantiates, refines and quantifies qualitative observations in regard to II in the spiking–bursting model which were initially made in [19]. The existence of lower bounds in spiking activity (characterized by $s_{1}$) required for positive “whole minus sum” II which was noticed in [19] is now expressed in the form of an explicit inequality (33) with the estimate (38) for the bound $s_{1}^{\min}$. The observation of [19] that typically $s_{1}^{\min}$ is mostly determined by burst probability and weakly depends upon time correlations of bursts also becomes supported by the quantitative result (33), (38). In particular, there is a range of spiking activity intensity $s_{1}≳0.17$, where the “whole minus sum” information is positive regardless of other system parameters, provided the spiking activity is spatially uncorrelated or negatively correlated across the system. When the burst probability is decreased (which implies less frequent activation of the astrocyte subsystem), the threshold value for spiking activity $s_{1}^{\min}$ also decreases.

We found that II scales as $\epsilon^{2}$, where ϵ is proportional (as per Equation (17)) to the Pearson’s time delayed correlation coefficient of the bursting component (which essentially characterizes the duration of bursts), for ϵ small (namely, within (36)), when other parameters (i.e., $p_{s}$ and spiking probability table $s_{x}$) are fixed. For the “whole minus sum” information, this is an analytical result. Note that the reasoning behind this result does not rely upon the assumption of spatial non-correlation of spiking activity (between bits), and thus applies generally to arbitrary spiking–bursting systems. According to a numerical calculation, this scaling approximately holds for the “decoder based” information as well.

Remarkably, II can not exceed the time delayed mutual information for the system as a whole, which in case of the spiking–bursting model in its present formulation is no greater than 1 bit.

The model provides a basis for possible modifications in order to apply integrated information concepts to systems exhibiting similar, but more complicated behavior (in particular, to neuronal [26,27,28,29] and neuron–astrocyte [24,30] networks). Such modifications might incorporate non-trivial spatial patterns in bursting, and causal interactions within and between the spiking and bursting subsystems.

The model can also be of interest as a new discrete-state test bench for different formalizations of integrated information, while available comparative studies of II measures mainly focus on Gaussian autoregressive models [17,18].

## Appendix A. Derivation of Parameters Scaling of the Spiking–Bursting Model

In order to formalize the reasoning in Section 4, we introduce an auxiliary 3-state process *W* with set of one-time states $\left\{s^{\prime },d,b\right\}$, where $s^{\prime }$ and *b* are always interpreted as spiking and bursting states in terms of Section 3, and *d* is another state, which is assumed to produce all bits equal 1 like in a burst, but in a time-uncorrelated manner (which is formalized by Equation (A4) below) like in a system-wide spike. When *W* is properly defined (by specifying all necessary probabilities, see below) and supplemented with a time-uncorrelated process *S* as a source of spontaneous activity for the state $s^{\prime }$, these together constitute a completely defined stochastic model {W,S}.

This 3-state based model may be mapped on equivalent (in terms of resultant realizations) 2-state based models as in Section 3 in an ambiguous way, because the state *d* may be equally interpreted either as a system-wide spike, or as a time-uncorrelated burst, thus producing two different dichotomous processes (which we denote as *V* and $V^{\prime }$) for the equivalent spiking–bursting models. The relationship between the states of *W*, *V* and $V^{\prime }$ is illustrated by the following diagram.

(A1)

As soon as *d*-states of *W* are interpreted in *V* as (spiking) *s*-states, the spontaneous activity process *S* accompanying *V* has to be supplemented with system-wide spikes whenever W=d, in addition to the spontaneous activity process $S^{\prime }$ for $V^{\prime }$. In order to maintain the absence of time correlations in spontaneous activity (which is essential for the analysis in Section 5), we assume time-uncorrelated choice between $W=s^{\prime }$ and W=d when V=s (which manifests below in Equation (A4)). Then the difference between the spontaneous components *S* and $S^{\prime }$ comes down to a difference in the corresponding one-time probability tables $s_{x}$ and $s_{x}^{\prime }$.

In the following, we proceed from the dichotomous process *V* defined as in Section 3, then define a consistent 3-state process *W*, and further obtain another dichotomous process $V^{\prime }$ for an equivalent model. Finally, we establish the relation between the corresponding probability tables of spontaneous activity $s_{x}$ and $s_{x}^{\prime }$.

The first dichotomous process *V* has states denoted by {s,b} and is related to *W* according to the rule V=s when $W=s^{\prime }$ or W=d, and V=b whenever W=b (see diagram (A1)). Assume fixed conditional probabilities

(A2a) $$\begin{array}{cc} p(W=s^{\prime }\;|\;V=s) & =\alpha , \end{array}$$

(A2b) $$\begin{array}{cc} p(W=d\;|\;V=s) & =\beta =1-\alpha , \end{array}$$

which implies one-time probabilities for *W* as

(A3) $$p_{s^{\prime }}=\alpha p_{s},\;p_{d}=\beta p_{s}.$$

The mentioned requirement of time-uncorrelated choice between $W=s^{\prime }$ and W=d when V=s is expressed by factorized two-time conditional probabilities

(A4a) $$\begin{array}{cc} p(W=s^{\prime }s^{\prime }\;|\;V=ss) & =\alpha^{2}, \end{array}$$

(A4b) $$\begin{array}{cc} p(W=s^{\prime }d\;|\;V=ss) & =\alpha \beta =p(W=ds^{\prime }\;|\;V=ss), \end{array}$$

(A4c) $$\begin{array}{cc} p(W=dd\;|\;V=ss) & =\beta^{2}. \end{array}$$

Given the two-time probability table for *V* (8) along with the conditional probabilities (A2) and (A4), we arrive at a two-time probability table for *W*

(A5)

Note that (A5) is consistent both with (A3), which is obtained by summation along the rows of (A5), and with (8), which is obtained by summation within the line-separated cell groups in (A5):

(A6a) $$\begin{array}{cc} p_{ss} & \equiv p_{s^{\prime }s^{\prime }}+p_{s^{\prime }d}+p_{ds^{\prime }}+p_{dd} \end{array}$$

(A6b) $$\begin{array}{cc} p_{sb} & \equiv p_{s^{\prime }b}+p_{db} \end{array}$$

(A6c) $$\begin{array}{cc} p_{bs} & \equiv p_{bs^{\prime }}+p_{bd} \end{array}$$

(A6d) $$\begin{array}{cc} p_{bb} & \equiv p_{bb}. \end{array}$$

Consider the other dichotomous process $V^{\prime }$ with states $\left\{s^{\prime },b^{\prime }\right\}$ obtained from *W* according to the rule $V^{\prime }=b^{\prime }$ when W=d or W=b, and $V^{\prime }=s^{\prime }$ whenever $W=s^{\prime }$ (see diagram (A1)). The two-time probability table for $V^{\prime }$ is obtained by another partitioning of the table (A5)

(A7)

with subsequent summation of cells within groups, which yields

(A8a) $$\begin{array}{cc} p_{s^{\prime }s^{\prime }} & =\alpha^{2}p_{ss}, \end{array}$$

(A8b) $$\begin{array}{cc} p_{s^{\prime }b^{\prime }} & =\alpha \left(\beta p_{ss}+p_{sb}\right)=p_{b^{\prime }s^{\prime }}, \end{array}$$

(A8c) $$\begin{array}{cc} p_{b^{\prime }b^{\prime }} & =\beta^{2}p_{ss}+2\beta p_{sb}+p_{bb}. \end{array}$$

The corresponding one-time probabilities for $V^{\prime }$ read

(A9a) $$\begin{array}{cc} p_{s^{\prime }} & =\alpha p_{s}, \end{array}$$

(A9b) $$\begin{array}{cc} p_{b^{\prime }} & =\beta p_{s}+p_{b}. \end{array}$$

In order to establish the relation between the one-time probability tables of spontaneous activity $s_{x}$ and $s_{x}^{\prime }$, we equate the resultant one-time probabilities of observing a given state *x* as per (6) for the two equivalent models {V,S} and $\left\{V^{\prime },S^{\prime }\right\}$

(A10a) $$\begin{array}{cc} p(x\neq 1) & =p_{s}s_{x}=p_{s^{\prime }}s_{x}^{\prime }, \end{array}$$

(A10b) $$\begin{array}{cc} p(x=1) & =p_{s}s_{1}+p_{b}=p_{s^{\prime }}s_{1}^{\prime }+p_{b^{\prime }}. \end{array}$$

Taking into account (A9), we finally get

(A11a) $$\begin{array}{cc} s_{x\neq 1} & =\alpha s_{x\neq 1}^{\prime }, \end{array}$$

(A11b) $$\begin{array}{cc} 1-s_{1} & =\alpha (1-s_{1}^{\prime }). \end{array}$$

Equations (A8), (A9) and (A11) fully describe the transformation of the spiking–bursting model which keeps the resultant stochastic process invariant by the construction of the transform. Taking into account that the dichotomous process is fully described by just two independent quantities, e.g., $p_{s}$ and $p_{ss}$, all other probabilities being expressed in terms of these due to normalization and stationarity, the full invariant transformation is uniquely identified by a combination of (A11a,b), (A8a) and (A9a), which together constitute the scaling (12).

Note that parameter α within its initial meaning (A2) may take on values in the range 0<α≤1 (case α=1 producing the identical transform). That said, in terms of the scaling (12a-d), all values α>0 are equally possible, so that mutually inverse values $\alpha =\alpha_{1}$ and $\alpha =\alpha_{2}=1/\alpha_{1}$ produce mutually inverse transforms.

## Appendix B. Expressing Mutual Information for the Spiking–Bursting Process

One-time entropy $H_{x}$ for the spiking–bursting process is expressed by (2) with probabilities p(x) taken from (6):

(A12) $$H_{x}=\sum_{x}\left\{p\left(x\right)\right\}=\sum_{x}\left\{p_{s}s_{x}\right\}+\left\{p_{1}\right\}-\left\{p_{s}s_{1}\right\},$$

where the additional terms besides the sum over *x* account for the specific expression (6b) for p(x=1). Using the relation

(A13) {ab}≡a{b}+{a}b,

which is derived directly from (19), and collecting similar terms, we arrive at

(A14) $$H_{x}=p_{s}H_{s}-p_{s}\left\{s_{1}\right\}+\left(1-s_{1}\right)\left\{p_{s}\right\}+\left\{p_{1}\right\},$$

where $H_{s}$ is the entropy of the spiking component taken alone

(A15) $$H_{s}=\sum_{x}\left\{s_{x}\right\}.$$

Two-time entropy is expressed similarly, by substituting probabilities p(xy) from (11) into the definition of entropy and taking into account the special cases with x=1 and/or y=1:

(A16) $$\begin{array}{cc} H_{xy}=\sum_{xy}\left\{p\left(xy\right)\right\}=\sum_{xy}\left\{p_{ss}s_{x}s_{y}\right\}\; & -\sum_{x}\left\{p_{ss}s_{x}s_{1}\right\}+\sum_{x}\left\{\pi s_{x}\right\}\\ \; & -\sum_{y}\left\{p_{ss}s_{1}s_{y}\right\}+\sum_{y}\left\{\pi s_{y}\right\}+\left\{p_{ss}s_{1}^{2}\right\}-2\left\{\pi s_{1}\right\}+\left\{p_{11}\right\}. \end{array}$$

Further, applying (A13) and using the notation (A15), we find

(A17a) $$\begin{array}{cc} \sum_{xy}\left\{p_{ss}s_{x}s_{y}\right\} & =p_{ss}\sum_{xy}\left\{s_{x}s_{y}\right\}+\left\{p_{ss}\right\}\sum_{xy}s_{x}s_{y}\\ \; & =p_{ss}\cdot 2H_{s}+\left\{p_{ss}\right\}, \end{array}$$

where we used the reasoning that $\sum_{xy}\left\{s_{x}s_{y}\right\}$ is the two-time entropy of the spiking component taken alone, which is (due to the postulated absence of time correlations in it) twice the one-time entropy $H_{s}$ (this of course can equally be found by direct calculation). Similarly, we get

(A17b) $$\begin{array}{cc} \sum_{x}\left\{p_{ss}s_{x}s_{1}\right\} & =p_{ss}s_{1}\sum_{x}\left\{s_{x}\right\}+\left\{p_{ss}s_{1}\right\}\sum_{x}s_{x}\\ \; & =p_{ss}s_{1}H_{s}+\left\{p_{ss}s_{1}\right\} \end{array}$$

and exactly the same expression for $\sum_{y}\left\{p_{ss}s_{1}s_{y}\right\}$, and also

(A17c) $$\begin{array}{cc} \sum_{y}\left\{\pi s_{y}\right\}=\sum_{x}\left\{\pi s_{x}\right\} & =\pi \sum_{x}\left\{s_{x}\right\}+\left\{\pi \right\}\sum_{x}s_{x}\\ \; & =\pi H_{s}+\left\{\pi \right\}. \end{array}$$

Substituting (A17a–c) into (A16), using (A13) where applicable, and collecting similar terms with the relation

(A18) $$p_{ss}+\pi -p_{ss}s_{1}\equiv p_{s}$$

taken into account, we arrive at

(A19) $$H_{xy}=2p_{s}H_{s}+{\left(1-s_{1}\right)}^{2}\left\{p_{ss}\right\}-2p_{s}\left\{s_{1}\right\}+2\left(1-s_{1}\right)\left\{\pi \right\}+\left\{p_{11}\right\}.$$

Finally, the expression (18) for mutual information is obtained by inserting (A14) and (A19) into the definition (1), with stationarity $H_{y}=H_{x}$ taken into account.

## Appendix C. Expanding I 0 in Powers of ϵ

Taylor series expansion for a function f(x) up to the quadratic term reads

(A20) $$f\left(x_{0}+\xi \right)=f\left(x_{0}\right)+f^{\prime }\left(x_{0}\right)\xi +f^{\prime \prime }\left(x_{0}\right)\frac{\xi^{2}}{2}+R\left(\xi \right).$$

The remainder term R(ξ) can be represented in the Lagrange’s form as

(A21) $$R\left(\xi \right)=f^{\prime \prime \prime }\left(c\right)\frac{\xi^{3}}{6},$$

where *c* is an unknown real quantity between $x_{0}$ and $x_{0}+\xi$.

The function f(x) can be approximated by omitting R(ξ) in (A20) if R(ξ) is negligible compared to the quadratic term, for which it is sufficient that

(A22a) $$\left|f^{\prime \prime \prime }\left(c\right)\frac{\xi^{3}}{6}\right|\ll \left|f^{\prime \prime }\left(x_{0}\right)\frac{\xi^{2}}{2}\right|$$

for any *c* between $x_{0}$ and $x_{0}+\xi$, namely, for

(A22b) $$c\in \left\{\begin{array}{cc} (x_{0},x_{0}+\xi ), & \mathrm{if}\;\xi >0,\\ (x_{0}-|\xi |,x_{0}), & \mathrm{if}\;\xi <0. \end{array}\right.$$

Consider the specific case

(A23) f(x)=−xlogx,x>0,

for which we get

(A24) $$f^{\prime }\left(x\right)=-\log x-1,\;f^{\prime \prime }\left(x\right)=-\frac{1}{x},\;f^{\prime \prime \prime }\left(x\right)=\frac{1}{x^{2}}.$$

As long as $f^{\prime \prime \prime }\left(x\right)$ is a falling function for any x>0, fulfilling (A22a) at the left boundary of (A22b) (at $c=x_{0}$ if ξ>0, and at $c=x_{0}-\left|\xi \right|$ if ξ<0) makes sure (A22a) is fulfilled in the whole interval (A22b). Precisely, the requirement is

(A25a) $$\begin{array}{cc} \left|\frac{1}{x_{0}^{2}}\frac{\xi^{3}}{6}\right| & \ll \left|\frac{1}{x_{0}}\frac{\xi^{2}}{2}\right|,\;\mathrm{if}\;\xi >0, \end{array}$$

(A25b) $$\begin{array}{cc} \left|\frac{1}{(x_{0}-{\left|\xi |\right)}^{2}}\frac{\xi^{3}}{6}\right| & \ll \left|\frac{1}{x_{0}}\frac{\xi^{2}}{2}\right|,\;\mathrm{if}\;\xi <0, \end{array}$$

which in the case ξ>0 reduces to

(A26) $$\frac{\xi }{3x_{0}}\ll 1,$$

and in the case ξ<0 to

(A27a) $$\frac{1}{3}\Phi \left(\frac{\left|\xi \right|}{x_{0}}\right)\ll 1,$$

where

(A27b) $$\Phi \left(\zeta \right)=\frac{\zeta }{{\left(1-\zeta \right)}^{2}}.$$

Replacing Φ(·) in (A27a) by its linearization Φ(ζ)≈ζ for small ζ, we reduce both (A26) and (A27a) to a single condition

(A28) $$\left|\xi \right|\ll 3x_{0}.$$

We use these considerations to expand in powers of ϵ the function $I_{0}\left(p_{s},\epsilon \right)$ defined in (21) with $p_{ss}$, $p_{sb}$, $p_{bb}$ substituted by their expressions in terms of ϵ according to (14). We note that the braces notation {·} defined in (19) is expressed via the function f(x) from (A23) as

(A29) $$\left\{q\right\}=\frac{f(q)}{\log2}.$$

Expanding this way the subexpressions of (21)

(A30a) $$\begin{array}{cc} \left\{p_{ss}\right\} & =\{p_{s}^{2}+\epsilon p_{s}^{2}\}, \end{array}$$

(A30b) $$\begin{array}{cc} \left\{p_{sb}\right\} & =\{p_{s}p_{b}-\epsilon p_{s}^{2}\}, \end{array}$$

(A30c) $$\begin{array}{cc} \left\{p_{bb}\right\} & =\{p_{b}^{2}+\epsilon p_{s}^{2}\}, \end{array}$$

we find by immediate calculation that the zero-order and linear in ϵ terms vanish, and the quadratic term yields (35). The condition (A28) has to be applied to all three subexpressions (A30a–c). Omitting the insignificant factor 3 in (A28), we obtain the applicability conditions

(A31a) $$\begin{array}{cc} |\epsilon p_{s}^{2}| & \ll p_{s}^{2}, \end{array}$$

(A31b) $$\begin{array}{cc} |\epsilon p_{s}^{2}| & \ll p_{s}p_{b}, \end{array}$$

(A31c) $$\begin{array}{cc} |\epsilon p_{s}^{2}| & \ll p_{b}^{2}, \end{array}$$

which is equivalent to

(A32a) $$\begin{array}{cc} \left|\epsilon \right| & \ll 1, \end{array}$$

(A32b) $$\begin{array}{cc} \left|\epsilon \right| & \ll \frac{p_{b}}{p_{s}}=\epsilon_{\max}, \end{array}$$

(A32c) $$\begin{array}{cc} \left|\epsilon \right| & \ll \epsilon_{\max}^{2}, \end{array}$$

where the notation $\epsilon_{\max}$ from (16) is used. We note that when $\epsilon_{\max}<1$, the condition (A32c) is the strongest among (A32a–c); when $\epsilon_{\max}>1$, the condition (A32a) is the strongest. Therefore, in both cases (A32b) can be dropped, thus producing (36).
